# New insights into molecular characterization and genetic diversity of *Eimeria* coccidian parasites in bats from diverse geographical regions of Thailand using nanopore-based DNA metabarcoding

**DOI:** 10.1016/j.crpvbd.2025.100327

**Published:** 2025-10-13

**Authors:** Chatchapon Sricharoensuk, Pathamet Khositharattanakool, Puckavadee Somwang, Supaporn Wacharapluesadee, Padet Siriyasatien, Kanok Preativatanyou

**Affiliations:** a Medical Parasitology Program, Department of Parasitology, Faculty of Medicine, Chulalongkorn University, Bangkok, 10330, Thailand; b School of Medicine, Mae Fah Luang University, Chiang Rai, 57100, Thailand; c Biomedical Technology Research Group for Vulnerable Populations, Mae Fah Luang University, Chiang Rai, 57100, Thailand; d Thai Red Cross Emerging Infectious Diseases Clinical Center, King Chulalongkorn Memorial Hospital, Bangkok, 10330, Thailand; e Center of Excellence in Vector Biology and Vector-Borne Disease, Chulalongkorn University, Bangkok, 10330, Thailand; f Department of Parasitology, Faculty of Medicine, Chulalongkorn University, Bangkok, 10330, Thailand

**Keywords:** *Eimeria*, Coccidia, Bats, Rodents, Small subunit ribosomal RNA gene, Genetic diversity

## Abstract

Bats represent over 1400 species globally, accounting for approximately one-fifth of all mammalian diversity, yet their gastrointestinal parasite communities remain understudied. Among these parasites, the genus *Eimeria* is one of the most commonly documented coccidian groups infecting bats. To date, more than 40 *Eimeria* spp. have been described from bats worldwide; however, molecular data are limited, and their evolutionary relationships with congeners infecting other vertebrate hosts remain largely unresolved. This study aims to elucidate the evolutionary connections between *Eimeria* parasites infecting bats and rodents, addressing a key question about shared ancestry and host-switching events across deeply divergent hosts. We investigated the genetic diversity and phylogenetic relationships of *Eimeria* spp. infecting Thai bats. Ninety-six genomic DNA samples extracted from bat guano, collected across six geographically distinct sites in prior research, were screened using SSU rRNA-PCR and nanopore amplicon sequencing. Host identification based on vertebrate *cox*1 gene sequencing revealed seven bat species (*Pteropus lylei*, *Taphozous melanopogon*, *Cynopterus brachyotis*, *Eonycteris spelaea*, *Mops plicatus*, *Hipposideros armiger*, and *Pteropus vampyrus*) along with the *Hipposideros larvatus* species complex. Haplotype network construction and phylogenetic analyses using Bayesian inference and maximum likelihood identified five putative genetic clusters of *Eimeria* sequences, including novel Haplogroups 1 and 2 that formed clearly distinct groups. The remaining three clusters showed close genetic affinities to known *Eimeria* species from bats (*Eimeria* sp. Bat10 and *Eimeria* sp. Bat31) and rodents (*E. ferrisi*). Notably, despite the deep evolutionary divergence between bats and rodents, *Eimeria* parasites infecting these hosts did not form entirely separate clades. Both phylogenies consistently revealed the polyphyletic nature of bat-derived *Eimeria* species, with multiple independent lineages interspersed among rodent taxa. This pattern supports hypotheses of shared ancestry or host-switching events, highlighting the complex evolutionary dynamics shaping *Eimeria* diversity across vertebrate hosts. The widespread distribution and genetic patterns observed in Haplogroup 1 suggest a recent population expansion potentially driven by ecological adaptability and host range dynamics. By focusing on the evolutionary relationships between bat and rodent *Eimeria*, this study advances our understanding of *Eimeria* diversity and host-parasite coevolution, emphasizing the importance of integrative molecular approaches in unravelling parasite evolutionary history across vertebrate taxa.

## Highlights

• The first molecular identification and assessment of the genetic diversity of bat-associated *Eimeria* parasites in Thailand.
• The overall prevalence of *Eimeria* spp. infection in bats was 70.8%.
• Twenty *Eimeria* haplotypes detected were categorized into five haplogroups, representing five putative genetic clusters.
• Haplogroup 1 was the most dominant, with a broad host range and widespread distribution.
• Phylogenetic analyses revealed bat-derived *Eimeria* as polyphyletic, suggesting shared ancestry and host-switching.

## Graphical abstract

**Figure:**
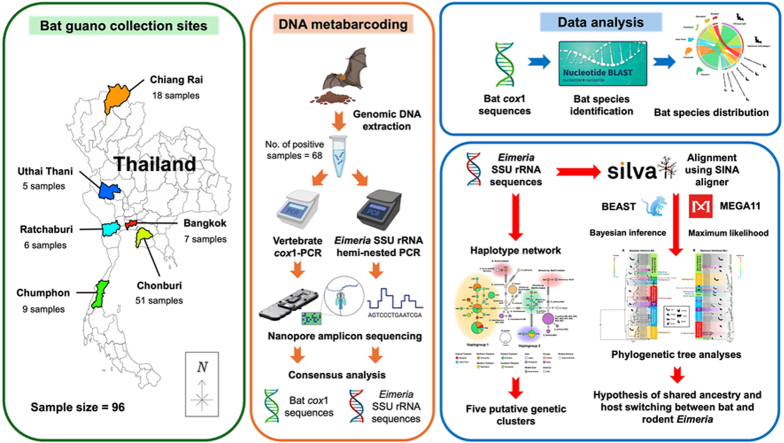

## 1 Introduction

Bats are known to harbor various gastrointestinal parasites, including protozoans of the genus *Eimeria*, which are intracellular coccidian parasites primarily infecting the epithelial cells of the intestinal tract (de Santana Miglionico et al., 2020). Species of *Eimeria* possess a direct life cycle, completing all developmental stages within a single host before releasing environmentally resistant oocysts through feces, facilitating fecal-oral transmission (Duszynski and Upton, 2001; Burrell et al., 2020). Although bats represent one of the most diverse mammalian orders, with over 1400 species worldwide (Wilson et al., 2019), documented *Eimeria* species in bats remain relatively few, with approximately 40 species described to date (de Santana Miglionico et al., 2020). In comparison, their rodent hosts support a significantly higher diversity of *Eimeria*, exceeding 400 species (Duszynski and Upton, 2001). This disparity highlights major gaps in our understanding of *Eimeria* diversity and host specificity in bats, warranting further investigation to elucidate their ecological and evolutionary relationships.

Traditionally, *Eimeria* spp. detection has relied on microscopic examination of oocysts in fecal specimens, with species identification primarily based on oocyst morphology and host specificity (Levine, 1982; Duszynski and Wilber, 1997). More recently, molecular techniques such as PCR and nucleotide sequencing have been applied to detect and identify *Eimeria* spp. in various vertebrate hosts (Kokuzawa et al., 2013; Kvičerová and Hypša, 2013; Mácová et al., 2018), including bats (Afonso et al., 2014; Murakoshi et al., 2016, 2018; Couso-Pérez et al., 2022). Although *Eimeria* species are generally considered stenoxenous with high host specificity, several studies have reported remarkable genetic similarity between *Eimeria* species infecting bats and those infecting rodents (Zhao et al., 2001; Afonso et al., 2014; Murakoshi et al., 2016; Couso-Pérez et al., 2022). These findings prompt a reevaluation of the strict host specificity paradigm and suggest complex evolutionary dynamics, including possible ancestral lineage sharing and ecological factors influencing host-parasite associations across divergent mammalian hosts.

However, current understanding is limited by the scarce molecular data available from bat-associated *Eimeria* and the lack of comprehensive phylogenetic analyses across diverse host species. Thai bats, with their high species diversity and varied habitats, offer a valuable opportunity to expand this dataset and enhance knowledge of *Eimeria* evolution across vertebrate hosts. By generating new molecular data from *Eimeria* infecting Thai bats, this study aims to improve understanding of parasite diversity and evolutionary relationships within a broader vertebrate context, thereby filling important gaps in our knowledge of *Eimeria* spp. evolution and host specificity.

To date, at least 148 bat species belonging to 11 families have been recorded in Thailand (Karapan et al., 2023). Given this high diversity and the substantial gaps in knowledge concerning coccidian parasites, no previous studies have investigated *Eimeria* species in Thai bats. Building on the limited available molecular data, the present study investigates the molecular occurrence, phylogenetics, and haplotype diversity of *Eimeria* parasites circulating in Thai bats from diverse geographical origins for the first time. This was accomplished using SSU rRNA-PCR combined with nanopore-based amplicon sequencing and metabarcoding analysis. Additionally, the bat host species harboring these parasites were identified. The novel molecular data and genetic analyses generated here provide valuable insights into the genetic diversity and phylogenetic relationships of *Eimeria* parasites in Thai bats.

## 2 Materials and methods

### 2.1 Genomic DNA sample collection

**Fig. 1:**
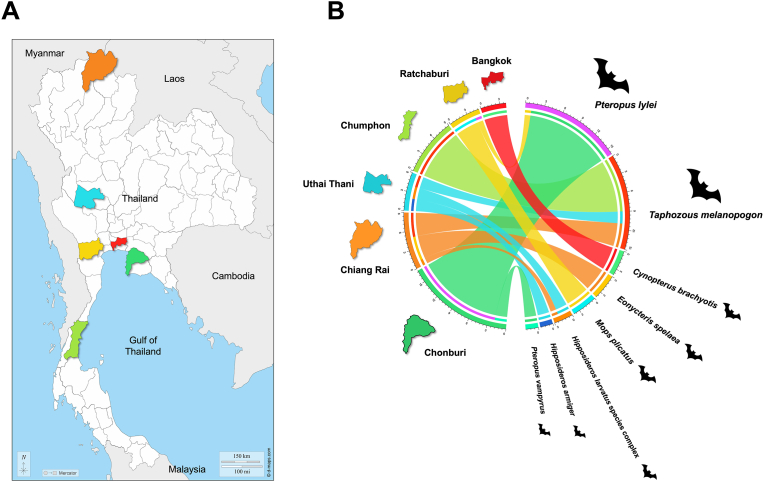
**A** Geographical locations of bat guano collection sites across Thailand. The Thailand map was sourced from the public domain (https://d-maps.com). **B** Circos plot illustrating the distribution of the bat host species identified across six study sites in this study.

This study utilized genomic DNA (gDNA) samples previously extracted during an earlier investigation screening for *Enterocytozoon bieneusi* in bat guano collected from diverse locations across Thailand (Savigamin et al., 2024). No new samples were obtained for the present analysis. Sampling sites, illustrated in Fig. 1, encompassed Bangkok (the capital), and five provinces representing different regions: Uthai Thani (central), Chiang Rai (northern), Chonburi (eastern), Ratchaburi (western), and Chumphon (southern). Guano specimens were collected from bat-inhabited caves within each province, except those from Bangkok, which were gathered from the Buddhadasa Indapanno Archives building.

The procedure for sample collection involved placing plastic containers on the floors beneath bat roosting sites to capture freshly deposited guano. These samples were collected after approximately 24 h and immediately preserved in 20 ml of Buffer AL lysis solution to stabilize nucleic acids. Genomic DNA extraction from these preserved samples was performed using the QIAamp Fast DNA Stool Mini Kit (Qiagen, Hilden, Germany), following the manufacturer’s protocol. The extracted DNA samples from the previous study provided the basis for the current investigation, which aimed to screen for *Eimeria* species.

### 2.2 Molecular screening of *Eimeria* parasites

*Eimeria* spp. were detected molecularly using a hemi-nested PCR targeting a hypervariable region of the SSU rRNA gene known for its sequence variability among *Eimeria* species, enabling species-level discrimination. Forward primer Ei18sF (5′-CCC AAT GAA AAC AGY TTC GAG G-3′) and reverse primer Ei18sR (5′-AAA CCC CCT ACT GTC GTT CTT G-3′) were used in the primary PCR, generating amplicons of approximately 550 bp. Secondary PCR was then performed using the forward primer Ei18sF and the reverse nested primer ER10 (5′-GCC CCC AAC TGT CCC TAT TA-3′), yielding products of approximately 444–447 bp (Couso-Pérez et al., 2019, 2022). The primary PCR mixture consisted of 2 μl of the DNA template, 0.3 μl of each 10 μM primer, and 5 μl of 2× KAPA HiFi HotStart ReadyMix, with nuclease-free water added to a total volume of 10 μl. For the secondary nested PCR, 2 μl of a 1:10 dilution of the first-round amplicons was used, combined with 0.75 μl of each 10 μM primer, 12.5 μl of 2× KAPA HiFi HotStart ReadyMix, and nuclease-free water to reach a total volume of 25 μl. Thermal cycling for both PCR rounds consisted of initial denaturation at 95 °C for 5 min, followed by 40 cycles of denaturation at 98 °C for 30 s, annealing at 56 °C for 30 s, and extension at 72 °C for 30 s, with a final extension step at 72 °C for 7 min. Positive controls consisted of gDNA previously confirmed to contain *Eimeria*, while nuclease-free water served as the negative control in all PCR runs to detect potential contamination. Nested PCR products were analyzed by electrophoresis on 1.5% (w/v) agarose gel stained with ethidium bromide and visualized using the GelDoc Go Imaging System (Bio-Rad, Hercules, CA, USA). PCR-positive SSU rRNA amplicons were subsequently purified using Agencourt AMPure XP beads (Beckman Coulter, CA, USA) for downstream sequencing.

### 2.3 Nanopore-based amplicon sequencing and metabarcoding analysis of bat-derived *Eimeria* species

To prepare the nanopore library, each purified amplicon underwent end-repairing and was ligated with native barcodes from the Native Barcoding Kit 96 V14 (Cat. No. SQK-NBD114.96; Oxford Nanopore Technologies, Oxford, UK) using the NEBNext Ultra II End Repair/dA-Tailing Module and the NEB Blunt/TA Ligase Master Mix (New England Biolabs, MA, USA). The barcoded samples were multiplexed and ligated to the sequencing adaptor using the NEBNext Quick Ligation Module (New England Biolabs, MA, USA). Sequencing was performed on a MinION® R10.4.1 flow cell. Super-accurate basecalling was conducted using Dorado (dna_r10.4.1_e8.2_400bps_sup@v.4.3.0 model), which integrates built-in quality controls, including primer and adapter detection, orientation determination, and internal filtering to ensure accurate sequence calls and minimize errors. Following demultiplexing and adaptor-barcode removal, sequenced reads with Q scores below 20 were filtered out using Chopper version 0.7.0 (De Coster and Rademakers, 2023). Only high-quality reads ranging from 300 to 500 bp were selected for consensus analysis. Accurate consensus sequences for each sample were generated using amplicon_sorter.py (version 2024_02_20) (Vierstraete and Braeckman, 2022). The resulting consensus sequences were then aligned against the GenBank database using BLASTn (Johnson et al., 2008). Taxonomic classification was performed using Megablast, an optimized BLASTn option designed for highly similar sequences, which utilizes sequence similarity and taxonomic data from the GenBank database to ensure accurate assignment. Given that the *Eimeria* sequences likely represent novel species with low similarity to known entries, no fixed percent identity threshold was applied. In this study, assignments were based on overall similarity patterns and phylogenetic context to accommodate potential new species diversity.

### 2.4 Identification of bat host species in *Eimeria*-positive gDNA samples

For bat host identification, each *Eimeria*-positive DNA sample was subjected to polymerase chain reaction targeting the cytochrome *c* oxidase subunit 1 (*cox*1) gene of vertebrate species. Vertebrate *cox*1-PCRs were performed using the primers VertCOI_7194_F (5′-CGM ATR AAY AAY ATR AGC TTC TGA Y-3′) and Mod_RepCOI_R (5′-TTC DGG RTG NCC RAA RAA TCA-3′), yielding a product of approximately 440 bp (Reeves et al., 2018). The PCR reaction consisted of 12.5 μl of 2× KAPA HiFi HotStart ReadyMix, 0.75 μl of each 10 μM primer, 3 μl of DNA template, and nuclease-free water, for a total volume of 25 μl. The thermocycling conditions were as follows: an initial denaturation step at 95 °C for 3 min, followed by 40 cycles of denaturation at 98 °C for 20 s, annealing at 48.5 °C for 30 s, extension at 72 °C for 1 min, with a final extension step at 72 °C for 7 min. The PCR products were verified in 1.5% (w/v) agarose gel electrophoresis with staining and visualization, as previously described.

Positive amplicons were purified and used for nanopore library preparation. Native barcoding, adaptor ligation, sample loading, and sequencing on the MinION® platform were performed as described previously, followed by super-accurate basecalling and metabarcoding analysis. The resulting consensus sequences were aligned to reference sequences in the GenBank database for taxonomic assignment using Megablast, optimized for highly similar sequences. Species-level identification of bats was assigned at a similarity threshold of ≥ 98%.

### 2.5 Haplotype network construction and genetic diversity analysis of *Eimeria* SSU rRNA gene sequences

Haplotype network analysis was performed to visualize the genetic diversity of *Eimeria* SSU rRNA gene haplotypes from this study and GenBank. Both single-nucleotide variations and small insertions and deletions (indels) were included as informative genetic differences. Gaps resulting from indels in the aligned sequences were considered evolutionary changes and incorporated into genetic distance calculations. A minimum-spanning network was generated using R version 4.4.0 (R Core Team, 2024) and RStudio version 2024.04.2+764 (Posit Team, 2025). Briefly, the SSU rRNA gene sequences in FASTA format were transformed into a DNA binary file necessary for network construction using the *adegenet* package, version 2.1.10 (Jombart, 2008). The *pegas* package, version 1.3, was then used to build the haplotype network using a list of DNA sequences in DNA binary format (Paradis, 2010). The ‘haplotype’ function in the *pegas* package was utilized to compare the DNA sequences, and each unique sequence was assigned to a distinct haplotype. Pairwise distances between haplotypes were then calculated using the ‘dist.dna’ function. A randomized minimum spanning tree was constructed based on the pairwise distances using the ‘rmst’ function. Finally, the ‘plot’ function was used to transform the randomized minimum spanning tree into a haplotype network.

Genetic diversity parameters, including the number of haplotypes, haplotype diversity (*Hd*), nucleotide diversity (*π*), the average number of nucleotide differences (*κ*), and Tajima’s *D* statistics, were calculated using the package *pegas* version 1.3. The *PopGenome* package, version 2.7.5 (Pfeifer et al., 2014), was used to calculate Fu and Li’s *D* statistics (Fu and Li, 1993). *P*-values lower than 0.05 were considered statistically significant.

### 2.6 Sequence alignment and phylogenetic inference of *Eimeria* SSU rRNA gene sequences

All haplotype sequences from this study, along with *Eimeria* spp. sequences previously identified in bats, rodents, and other vertebrate hosts and retrieved from the GenBank database, were aligned using SINA aligner version 1.2.12 (Pruesse et al., 2012). This tool performs reference-based sequence alignment against the highly curated global SILVA SSU rRNA gene database, which incorporates secondary structure information to ensure that compensatory base changes in paired (stem) regions of the SSU rRNA gene are preserved. Default alignment parameters were used, including gap opening and extension penalties optimized specifically for rRNA sequences.

The aligned sequences were then subjected to phylogenetic analyses using Bayesian inference (BI) and maximum likelihood (ML) methods. The best-fitting evolutionary model for BI was selected with ModelFinder (Kalyaanamoorthy et al., 2017) based on the lowest Bayesian Information Criterion (BIC) score, with TIM3+R2 identified as optimal for our data. The Bayesian phylogenetic tree was generated using BEAST version 2.7.7 (Bouckeart et al., 2019), with posterior probabilities calculated from 100,000,000 MCMC steps and the first 25% discarded as “burn-in”. For ML analysis, MEGA 11 software (Tamura et al., 2021) was used to select the best-fitting model (T92+G+I) and to construct a phylogenetic tree with 1000 bootstrap replicates. Both BI and ML trees were visualized using FigTree version 1.4.4 (Rambaut, 2018).

## 3 Results

### 3.1 Metabarcoding analysis of *Eimeria* spp. and host identification

A total of 96 bat guano samples were molecularly screened for *Eimeria* spp. DNA using a hemi-nested PCR targeting the SSU rRNA gene. Of these samples, 68 tested positive, resulting in an overall infection prevalence of 70.8%. Infection prevalence varied among locations: Bangkok (4/7, 57.1%), Uthai Thani (5/5, 100%), Chiang Rai (18/18, 100%), Ratchaburi (6/6, 100%), Chonburi (26/51, 51%), and Chumphon (9/9, 100%).

Nanopore-based amplicon sequencing produced 3,448,758 high-quality reads (Q scores ≥ 20) from positive samples, yielding 79 consensus SSU rRNA sequences from the 68 *Eimeria*-positive samples. BLASTn comparison against GenBank sequences revealed that most *Eimeria* sequences were closely related to *E. rioarribaensis* (GenBank: AF307877), with percent identities ranging from 97.1% to 98.0%. Several sequences matched *Eimeria* isolates from Philippine bats, such as *Eimeria* sp. Bat10 (GenBank: LC089986) and *Eimeria* sp. Bat31 (GenBank: LC089983), exhibiting higher similarity (98.9–99.6%). Notably, a subset of sequences showed closer affinity to *Eimeria* species infecting non-bat hosts: *Eimeria* sp. (GenBank: MH349726) from the Philippine tarsier (*Carlito syrichta*) with identities between 94.4% and 94.6%, and *Eimeria ferrisi* (GenBank: MH751961) from the house mouse (*Mus musculus*) exhibiting 99.8% identity.

Host identification using vertebrate *cox*1-PCR followed by MinION® sequencing was successful in 46 *Eimeria*-positive guano samples collected across six locations: Bangkok (*n* = 4), Uthai Thani (*n* = 4), Chiang Rai (*n* = 7), Ratchaburi (*n* = 5), Chonburi (*n* = 17), and Chumphon (*n* = 9). The bats were molecularly assigned to seven species and one species complex across six genera: lesser short-nosed fruit bat (*Cynopterus brachyotis*), great roundleaf bat (*Hipposideros armiger*), black-bearded tomb bat (*Taphozous melanopogon*), cave nectar bat (*Eonycteris spelaea*), wrinkle-lipped free-tailed bat (*Mops plicatus*), Lyle’s flying fox (*Pteropus lylei*), large flying fox (*Pteropus vampyrus*), and the *Hipposideros larvatus* species complex. These taxa showed high genetic identities (99.5–100%) to GenBank reference sequences, except the *Hipposideros larvatus* species complex (98.2%). Non-redundant *cox*1 sequences of identified bat species were deposited in the GenBank database under the accession numbers PX169707-PX169727. The geographical distribution of these bat hosts is illustrated in Fig. 1. Table 1 summarizes the prevalence of *Eimeria* infection, associated bat hosts, and closest reference sequences with identity scores for the study sites.

**Table 1:** Prevalence of *Eimeria* infection across six provinces in Thailand, identified bat hosts, and the closest *Eimeria* reference sequences in the GenBank database.

Localities and geographical coordinates	Collection date	*n*/*N* (%)	Host species identified in *Eimeria-*positive samples	Closest reference sequence in GenBank (accession number and associated host) and corresponding percent identity^a^
Bangkok (Central) [13°48′59.4″N, 100°33′29.4″E]	January 2023	4/7 (57.1)	*Cynopterus brachyotis* (*n* = 3)	*E. rioarribaensis* (AF307877, *Myotis ciliolabrum*), 97.7%
			*Cynopterus brachyotis* (*n* = 1)	*E. rioarribaensis* (AF307877), 97.5%
Uthai Thani (Central) [15°0′43.56″N, 99°26′36.203″E]	February 2023	5/5 (100)	*Hipposideros armiger + Hipposideros larvatus* species complex (*n* = 1)	*E. rioarribaensis* (AF307877), 98.0%
				*E. rioarribaensis* (AF307877), 97.7%
			*Hipposideros armiger + Hipposideros larvatus* species complex (*n* = 1)	*Eimeria* sp. Bat10 (LC089986, *Rhinolophus inops*), 99.3%
				*Eimeria* sp. Bat10 (LC089986), 99.6%
			*Taphozous melanopogon* (*n* = 1)	*E. rioarribaensis* (AF307877), 97.8%
				*E. rioarribaensis* (AF307877), 97.7%
			*Taphozous melanopogon* (*n* = 1)	*E. rioarribaensis* (AF307877), 97.3%
				*E. rioarribaensis* (AF307877), 97.7%
			NA (*n* = 1)	*E. rioarribaensis* (AF307877), 97.1%
				*Eimeria* sp. Bat10 (LC089986), 99.3%
Chiang Rai (Northern) [19°55′3.504″N, 99°47′20.076″E]	February 2023	18/18 (100)	*Eonycteris spelaea* (*n* = 2)	*E. rioarribaensis* (AF307877), 97.3%
			*Eonycteris spelaea* (*n* = 1)	*E. rioarribaensis* (AF307877), 97.7%
			*Eonycteris spelaea* + *Taphozous melanopogon* (*n* = 1)	*E. rioarribaensis* (AF307877), 98.0%
				*Eimeria* sp. Bat31 (LC089983, *Eonycteris spelaea*), 99.6%
			*Taphozous melanopogon* + *Hipposideros larvatus* species complex (*n* = 1)	*E. rioarribaensis* (AF307877), 98.0%
				*E. rioarribaensis* (AF307877), 97.7%
			*Taphozous melanopogon* (*n* = 1)	*E. rioarribaensis* (AF307877), 98.0%
			*Taphozous melanopogon* (*n* = 1)	*E. rioarribaensis* (AF307877), 97.7%
			NA (*n* = 4)	*E. rioarribaensis* (AF307877), 97.3%
			NA (*n* = 3)	*E. rioarribaensis* (AF307877), 98.0%
			NA (*n* = 2)	*E. rioarribaensis* (AF307877), 97.7%
			NA (*n* = 1)	*E. rioarribaensis* (AF307877), 97.5%
			NA (*n* = 1)	*E. ferrisi* (MH751961, *Mus musculus*), 99.8%
				*Eimeria* sp. Bat10 (LC089986), 98.9%
Ratchaburi (Western) [13°43′4.27″N, 99°46′16.539″E]	August 2022	6/6 (100)	*Mops plicatus* (*n* = 4)	*Eimeria* sp. in Philippine tarsier (MH349726, *Carlito syrichta*), 94.6%
			*Pteropus lylei* (*n* = 1)	*E. rioarribaensis* (AF307877), 98.0%
			NA (*n* = 1)	*Eimeria* sp. Bat10 (LC089986), 99.3%
Chonburi (Eastern) [13°30′19.8″N, 101°9′56.088″E]	July 2022	26/51 (51.0)	*Pteropus lylei* (*n* = 6)	*E. rioarribaensis* (AF307877), 97.7%
			*Pteropus lylei* (*n* = 3)	*E. rioarribaensis* (AF307877), 98.0%
			*Pteropus lylei* (*n* = 3)	*E. rioarribaensis* (AF307877), 97.5%
			*Pteropus lylei* (*n* = 1)	*Eimeria* sp. in Philippine tarsier (MH349726), 94.6%
			*Pteropus lylei* (*n* = 1)	*E. rioarribaensis* (AF307877), 98.0%
				*Eimeria* sp. in Philippine tarsier (MH349726), 94.6%
			*Pteropus lylei* (*n* = 1)	*E. rioarribaensis* (AF307877), 97.3%
				*Eimeria* sp. in Philippine tarsier (MH349726), 94.6%
			*Pteropus vampyrus* (*n* = 1)	*E. rioarribaensis* (AF307877), 97.5%
			*Pteropus vampyrus* (*n* = 1)	*E. rioarribaensis* (AF307877), 97.7%
				*Eimeria* sp. in Philippine tarsier (MH349726), 94.4%
			NA (*n* = 5)	*E. rioarribaensis* (AF307877), 97.5%
			NA (*n* = 4)	*E. rioarribaensis* (AF307877), 97.7%
Chumphon (Southern) [10°37′25.572″N, 99°6′47.447″E]	January 2023	9/9 (100)	*Taphozous melanopogon* (*n* = 5)	*E. rioarribaensis* (AF307877), 97.7%
			*Taphozous melanopogon* (*n* = 3)	*E. rioarribaensis* (AF307877), 97.3%
			*Taphozous melanopogon* (*n* = 1)	*E. rioarribaensis* (AF307877), 98.0%

*Abbreviations*: *n*, number of positive samples; *N*, total number of samples; NA, not amplified.

a Multiple sequence variants within the same host species may share the same reference sequence and exhibit identical percent identity.

### 3.2 Haplotype diversity and neutrality statistics of *Eimeria* circulating in Thai bats

A haplotype network was constructed to evaluate the genetic diversity and phylogeographic structure of *Eimeria* isolates from this study, alongside other bat *Eimeria* species and related rodent-infecting taxa. This analysis included a total of 130 SSU rRNA sequences: all 79 sequences obtained from bat guano samples in this study and 51 previously reported sequences, comprising 49 from other bat *Eimeria* species and 2 from rodent-related species, collected across diverse geographical regions (Supplementary Table S1). These regions include Thailand (the focus of this study), Japan (Murakoshi et al., 2018), the Philippines (Murakoshi et al., 2016) in Asia; Saudi Arabia in the Middle East (Mohammed et al., 2020); France (Afonso et al., 2014) and Spain (Couso-Pérez et al., 2022) in Europe; and the USA (Zhao et al., 2001). The network analysis identified 87 polymorphic sites across the dataset, which were assigned to 38 distinct haplotypes. Of these, 20 haplotypes (H01-H20) were detected in Thai bats and deposited in the GenBank database under the accession numbers PV738151-PV738170.

**Fig. 2:**
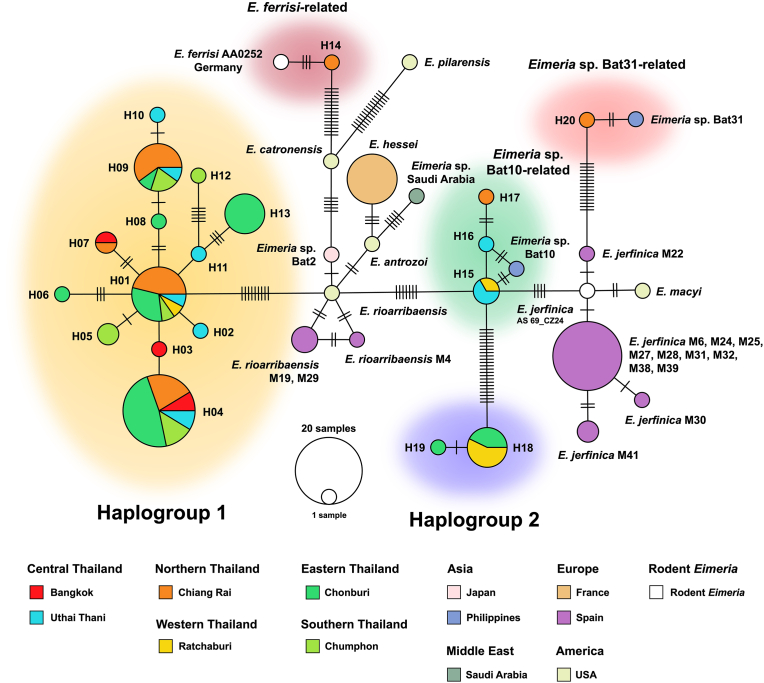
Haplotype diversity of *Eimeria* parasites in Thai bats and bats from various geographical regions, based on partial SSU rRNA sequences. The size of each circle corresponds to the number of unique haplotypes. Hatch marks along the connecting lines indicate the number of nucleotide polymorphic sites between haplotypes. Colored circles (excluding white) represent the geographical locations of the sampling sites. Five clusters, highlighted with colored shadow backgrounds, contain the haplotypes identified from bat guano samples in this study.

The haplotype network of *Eimeria* populations in Thai bats formed five distinct clusters, indicating that the parasites identified in this study belong to five separate genetic groups. As illustrated in Fig. 2, most haplotypes from this study (H01-H13) belong to Haplogroup 1, which forms a cluster connected to *E. rioarribaensis*, known to infect several bat species in different regions, including western small-footed bats (*Myotis ciliolabrum*) in North America (Duszynski et al., 1999), northern bats (*Eptesicus nilssonii*) in Japan (Murakoshi et al., 2018), and Mediterranean horseshoe bats (*Rhinolophus euryale*), greater mouse-eared bats (*Myotis myotis*), and greater noctule bats (*Nyctalus lasiopterus*) in Spain (Couso-Pérez et al., 2022). Haplogroup 1 exhibited high haplotype richness and strong geographical diversity, with shared haplotypes found across multiple provinces in Thailand, including Bangkok, Uthai Thani, Chiang Rai, Ratchaburi, Chonburi, and Chumphon. This haplogroup displays a star-like network pattern centered on haplotype H01, from which most members differ by only a few mutations. The common haplotypes H01, H04, and H09 are widely distributed across Thailand, indicating possible host generalism or widespread transmission.

Haplogroup 2 (H18 and H19) is connected to a cluster of *Eimeria* sp. Bat10-related haplotypes (H15-H17); however, these two clusters are genetically distant. Haplotype H20 is related to *Eimeria* sp. Bat31, which infects *Eonycteris spelaea* in the Philippines (Murakoshi et al., 2016). Both also connect with the cluster of rodent-infecting *E. jerfinica* (Mácová et al., 2018), also known to infect several bat species in Spain (Couso-Pérez et al., 2022). The distinct H14 haplotype, related to *E. ferrisi*, appears to have descended from *E. catronensis*, which infects little brown bats (*Myotis lucifugus*), Yuma myotis (*M. yumanensis*) (Scott and Duszynski, 1997), and northern long-eared bats (*M. septentrionalis*) (McAllister et al., 2004), through mutational steps.

The haplotype network also revealed transboundary relationships and a broader Asian distribution, including sequences from Japan and the Philippines. Notably, *Eimeria* sp. Bat10-related haplotypes connect closely with *E. jerfinica* and *E. macyi* sequences from Europe and the USA, supporting intercontinental connectivity among bat and rodent-associated *Eimeria* lineages. Overall, the network highlights a complex pattern of *Eimeria* diversity in bats and rodents, reflecting both localized transmission within Thailand and broader global biogeographical patterns.

Within the *Eimeria* population detected in bat guano samples, a total of 66 polymorphic sites were identified. Focusing on Haplogroup 1, which includes 13 distinct haplotypes, 15 polymorphic sites were observed. Haplotype diversity (*Hd*) was relatively high for both the overall population (0.8627 ± 0.0237) and Haplogroup 1 (0.8021 ± 0.0319), reflecting substantial genetic variation within these groups. In contrast, nucleotide diversity (*π*) was low, with values of 0.0218 ± 0.0112 for the entire population and 0.0042 ± 0.0027 for Haplogroup 1, indicating limited sequence divergence among haplotypes. Neutrality tests produced negative values for Tajima’s *D* and Fu and Li’s *D* across all groups, suggesting potential population expansion or purifying selection; however, none of these results were statistically significant (Table 2). These findings complement the haplotype network analysis, highlighting a genetically diverse but closely related *Eimeria* population circulating in Thai bats.

**Table 2:** Genetic diversity and neutrality statistics of *Eimeria* SSU rRNA gene haplotypes identified in bat guano samples in this study.

Population	Sample size	No. of haplotypes (*H*)	No. of polymorphic sites (*S*)	Average no. of nucleotide differences (*κ*)	Haplotype diversity (*Hd*)	Nucleotide diversity (*π*)	Tajima’s *D*	Fu and Li’s *D*
*Eimeria* spp. (total)	79	20	66	10.3463	0.8627 ± 0.0237	0.0218 ± 0.0112	−0.7473 (*P* = 0.45)	−1.3125 (*P* > 0.05)
Haplogroup 1	64	13	15	2.5407	0.8021 ± 0.0319	0.0042 ± 0.0027	−0.5872 (*P* = 0.56)	−0.9945 (*P* > 0.05)
Haplogroup 2	8	2	1	0.2500	0.2500 ± 0.1838	0.0006 ± 0.0008	−1.0548 (*P* = 0.29)	−1.1264 (*P* > 0.05)
*Eimeria* sp. Bat10-related	5	3	3	1.1000	0.7000 ± 0.2074	0.0018 ± 0.0018	−1.4854 (*P* = 0.13)	−0.9726 (*P* > 0.05)

### 3.3 Bayesian inference and maximum likelihood phylogenetic analysis of *Eimeria* across different hosts

Phylogenetic relationships among *Eimeria* spp. isolated from various vertebrate hosts, including bats, rodents, and others, were examined using both BI and ML methods. The analysis incorporated 20 distinct *Eimeria* haplotypes obtained in this study, together with 72 reference sequences from GenBank representing *Eimeria* species infecting rodents (*n* = 37), bats (*n* = 25), shrews (*n* = 3), chickens (*n* = 2), cattle (*n* = 2), rabbits (*n* = 2), and a tarsier (*n* = 1) (Supplementary Table S2). *Toxoplasma gondii* was included as the outgroup.

**Fig. 3:**
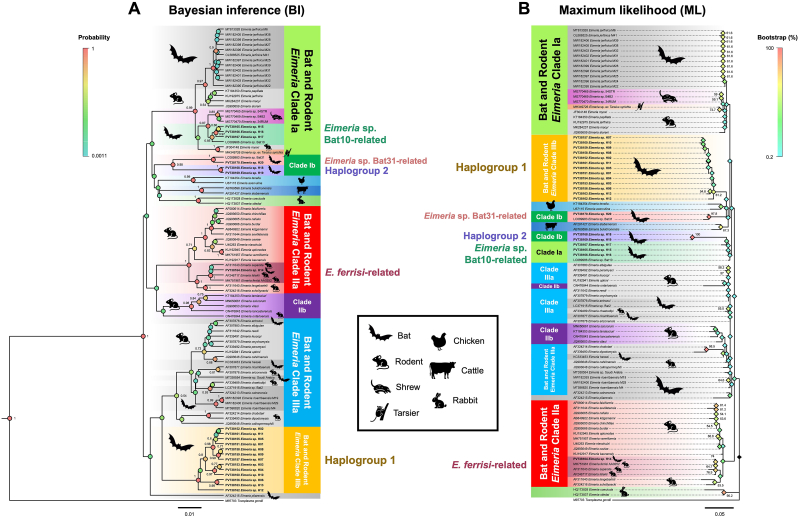
Phylogenetic relationships of *Eimeria* spp. isolated from bats, rodents, and other vertebrate hosts based on partial SSU rRNA sequences. Bayesian inference (BI) phylogeny (**A**) and maximum likelihood (ML) phylogeny (**B**) are shown. Major clades and haplogroups are highlighted with consistent color designations across both trees: Bat and Rodent *Eimeria* Clade Ia (*green*), Clade Ib (*dark green*), Clade IIa (*red*), Clade IIb (*purple*), Clade IIIa (*sky blue*), and Clade IIIb (*yellow*). Posterior probabilities (BI) and bootstrap support values (ML) are represented by node symbols colored according to the respective scale bars (left: posterior probability; right: bootstrap percentage), indicating statistical support levels. Host species are depicted by silhouettes adjacent to terminal branches, including bat, rodent, shrew, tarsier, chicken, cattle, and rabbit. *Toxoplasma gondii* was used as the outgroup in both analyses.

The BI phylogenetic tree (Fig. 3A) revealed a well-resolved topology with multiple strongly supported clades, as indicated by high posterior probability values (color-coded circles at nodes). Bat- and rodent-derived *Eimeria* sequences were widely distributed across three major clades: Clades Ia (green), IIa (red), and IIIa (sky blue). Clade Ia formed a cohesive ‘Bat and Rodent *Eimeria* Clade Ia’, with bat isolates clustering closely with rodent taxa. Similar patterns were observed in Clades IIa and IIIa, with bat and rodent lineages intermingled across these groups. Conversely, Clades Ib, IIb, and IIIb consisted predominantly of host-restricted isolates and were well separated from the main bat-rodent clades, likely reflecting host-specific adaptations.

Within these clades, two haplogroups containing bat-derived isolates were identified: Haplogroup 1 (yellow), associated with Clade IIIa, and Haplogroup 2 (purple), grouping within Clade Ib alongside *Eimeria* sp. Bat31-related isolates. Additionally, three haplotypes (H15-H17), linked to *Eimeria* sp. Bat10, clustered within Clade Ia, while the distinct H14 haplotype clustered with *E. ferrisi* isolates in Clade IIa, underscoring its taxonomic distinctness. Node support at major clades was strong, with posterior probabilities approaching 1.0, confirming the robustness of these relationships.

The ML phylogenetic tree (Fig. 3B) largely corroborated the BI phylogenetic tree, recovering the same major clades with high bootstrap support (up to 100%) at key nodes. Bat- and rodent-derived isolates were similarly distributed across Clades Ia, IIa, and IIIa, mirroring the polyphyletic and interspersed pattern observed in the BI analysis. 10.13039/100021088Clade Ia again emerged as a prominent group combining isolates from both hosts, with bootstrap support indicating moderate confidence. Clades IIa and IIIa also contained mixed host isolates, providing further evidence for multiple shared evolutionary lineages. Host-restricted clades Ib, IIb, and IIIb were consistently resolved in the ML tree, with Haplogroup 1 (yellow) linked to Clade IIIb and Haplogroup 2 (purple) closely related to *Eimeria* sp. Bat10 isolates, reflecting patterns seen in the BI tree. The distinct lineage represented by the haplotype H14 related to *E. ferrisi* was also moderately supported, suggesting a separate evolutionary lineage stemming from rodent-associated ancestors. Overall, both BI and ML analyses consistently revealed the polyphyletic nature of bat-derived *Eimeria* species, characterized by multiple independent lineages interspersed among rodent taxa.

## 4 Discussion

To date, 40 species of *Eimeria* have been morphologically described from 30 bat species across 18 genera and five families worldwide (de Santana Miglionico et al., 2020). Despite advances in understanding bat diversity, the diversity of bat-associated coccidian parasites in the family Eimeriidae remains poorly characterized. Molecular data are limited, with sequences for only six valid bat *Eimeria* species currently available in GenBank: *E. antrozoi* (Zhao et al., 2001), *E. catronensis*, *E. hessei* (Afonso et al., 2014), *E. macyi* (Miles et al., 2019), *E. pilarensis*, and *E. rioarribaensis* (Zhao et al., 2001). This scarcity of molecular information hinders clarification of the taxonomy and evolutionary relationships of bat *Eimeria* across different geographical regions.

In this study, we found a high prevalence of *Eimeria* infection (70.8%) among bat populations in Thailand, particularly in insectivorous species. This finding supports prior research that insectivorous bats are more likely to harbor *Eimeria* infections than frugivorous bats (Adhikari et al., 2020; Couso-Pérez et al., 2022). Using nanopore-based metabarcoding that targets the partial SSU rRNA gene, we uncovered complex mixed infections and extensive genetic diversity, which is often underestimated by traditional morphology-based and single-marker molecular approaches. This high-throughput sequencing approach provides greater resolution to distinguish morphologically similar but genetically distinct parasite lineages, revealing hidden parasite diversity and intricate host-parasite relationships in natural populations.

The SSU rRNA gene is a widely used molecular marker for *Eimeria* spp. detection due to its species-specific polymorphisms, allowing differentiation among closely related taxa. SSU rRNA-PCR combined with Sanger sequencing remains a highly sensitive tool for identifying *Eimeria* species and novel variants (Couso-Pérez et al., 2019, 2022; Chen et al., 2024). However, natural infections often involve multiple co-infecting *Eimeria* species (Carvalho et al., 2011; Bawm et al., 2020; Nguyen et al., 2021), complicating species identification by morphology and Sanger sequencing, which produces a single consensus sequence per sample and thus limits taxonomic resolution. Our use of nanopore-based metabarcoding overcomes these limitations by enabling simultaneous identification of multiple SSU rRNA sequences from the same guano sample, allowing accurate assessment of species composition and genetic diversity in bat *Eimeria* (Krehenwinkel et al., 2019; Wang et al., 2021).

Among the genetic clusters identified, Haplogroup 1 was widespread and dominant, occurring across multiple bat species and geographical locations. While this haplogroup is phylogenetically close to *E. rioarribaensis*, it is genetically distinct, with a genetic divergence of 2.0–2.9%, suggesting that Haplogroup 1 likely represents a distinct genetic cluster widely distributed in Thailand. Its star-like haplotype network, characterized by high haplotype diversity and low nucleotide diversity, indicates recent population expansion or diversification from a common ancestor, facilitating adaptation to diverse hosts and ecological niches (Ferreri and Qu, 2011; Li and Xiao, 2020).

In addition to Haplogroup 1, we identified Haplogroup 2 and haplotypes related to *Eimeria* sp. Bat10, Bat31, and *E. ferrisi*. The haplotypes H15, H16, H17, and H20 showed a close relationship with sequences from Philippine bats (*Eimeria* sp. Bat10 and Bat31), suggesting potentially widespread Southeast Asian lineages. The detection of an *E. ferrisi*-related haplotype (H14) in bat guano requires cautious interpretation, as *E. ferrisi* is primarily associated with the house mouse (*Mus musculus*) (Hofmannová et al., 2016; Jarquín-Díaz et al., 2019). Vertebrate *cox*1 metabarcoding confirmed the presence of only bat DNA in the samples, ruling out obvious contamination by rodent feces. However, since only a single *E. ferrisi*-related sequence was detected, this does not provide definitive evidence of genuine infection in bats. Potential spurious infections through coprophagous insect prey that may carry rodent *Eimeria* oocysts (Aizpurua et al., 2018; Alberdi et al., 2020; Tiede et al., 2020; Couso-Pérez et al., 2022), as well as environmental contamination, remain possible explanations. Given the usual high host specificity of *Eimeria* species, including *E. ferrisi*, cross-species infections are likely rare or transient. Confirmation of true infection would require histopathological evidence showing parasite development within bat tissues as well as consistent molecular detection across multiple specimens (Murakoshi et al., 2018). While *E. ferrisi* infection in rodent species has not yet been reported in Thailand, the detection of this rodent *Eimeria* species in our guano sample suggests that it is likely to exist within rodent populations in the country.

Phylogenetic analyses consistently reveal that bat- and rodent-derived *Eimeria* lineages are interspersed across Clades Ia, IIa, and IIIa, indicating a polyphyletic origin. Despite the deep evolutionary divergence between their hosts, bat-derived *Eimeria* do not form distinct, separate clades from those infecting rodents. This pattern supports previous hypotheses of either shared ancestry or host-switching events between bats and rodents (Zhao et al., 2001; Afonso et al., 2014; Murakoshi et al., 2016; Couso-Pérez et al., 2022). Such complex evolutionary relationships are likely driven by ecological factors, including food chains and shared habitats, which facilitate cross-species transmission of *Eimeria* parasites (Aizpurua et al., 2018; Alberdi et al., 2020).

The reliability of traditional morphological identification, which is based on oocyst characteristics and host specificity, is limited by overlapping morphologies (Duszynski, 1971; Long and Joyner, 1984; Nguyen et al., 2021), intraspecies variation, and cryptic diversity (Reshi et al., 2025). Molecular approaches are therefore pivotal for precise species delineation and phylogenetic inference. However, the conserved nature of SSU rRNA necessitates future multilocus strategies, combined with morphometric and histological data, to fully resolve taxonomy and transmission ecology. In addition to nuclear markers, mitochondrial cytochrome *c* oxidase subunit 1 gene (*cox*1) and plastid markers, such as 23S rDNA and open reading frame 470 (ORF470), have demonstrated potential in species discrimination and uncovering hidden genetic variation among *Eimeria* parasites (Zhao and Duszynski, 2001; Zhao et al., 2001; Cai et al., 2003; Jarquín-Díaz et al., 2020; Zhou et al., 2023). These organellar markers complement nuclear data by providing higher resolution phylogenies and improving species delineation, thereby advancing the study of *Eimeria* taxonomy and evolution.

Ultimately, this study provides the first molecular evidence of the widespread occurrence and genetic diversity of *Eimeria* coccidian parasites infecting multiple bat species across Thailand. However, these findings remain preliminary without integrated morphological characterization of corresponding sporulated oocysts. Accordingly, further research combining detailed morphometric and histopathological analyses with expanded molecular characterization of sporulated oocysts from a larger and more geographically extensive guano collection is essential to fully elucidate the diversity, taxonomy, and phylogenetic relationships of bat *Eimeria* in this region.

## 5 Conclusions

This study provides novel molecular insights into the diversity and distribution of *Eimeria* parasites infecting bats in Thailand. Using nanopore-based metabarcoding, five distinct genetic groups were identified, several of which exhibit close evolutionary relationships with rodent-associated *Eimeria*. The polyphyletic nature of bat-derived *Eimeria*, characterized by multiple independent lineages interspersed among rodent parasites, supports hypotheses of historical shared ancestry and recent host-switching events. The star-like haplotype network pattern of the dominant haplogroup in Thai bats suggests recent adaptive diversification and a possible expansion of host range. Future work will employ formal co-phylogenetic reconciliation analyses, integrating expanded multilocus genetic data and broader sampling, to thoroughly evaluate these hypotheses. While these findings significantly enhance our understanding of bat *Eimeria* diversity, comprehensive morphological and histopathological characterization remains essential to validate these genetic patterns and accurately delineate species boundaries within this parasite group.

## Ethical approval

The research design and methodology for specimen collection and molecular investigation in this study were reviewed and approved by the animal research ethics committee of Chulalongkorn University Animal Care and Use Protocol (COA No. 005/2565 and COE No. 001/2024), Faculty of Medicine, Chulalongkorn University, Bangkok, Thailand.

## CRediT authorship contribution statement

**Chatchapon Sricharoensuk:** Formal analysis, Investigation, Methodology, Visualization, Writing – original draft, Writing – review & editing. **Pathamet Khositharattanakool:** Investigation, Resources. **Puckavadee Somwang:** Investigation, Resources. **Supaporn Wacharapluesadee:** Investigation, Resources. **Padet Siriyasatien:** Funding acquisition, Investigation, Resources, Supervision, Validation. **Kanok Preativatanyou:** Conceptualization, Data curation, Formal analysis, Funding acquisition, Investigation, Methodology, Project administration, Resources, Supervision, Validation, Visualization, Writing – original draft, Writing – review & editing.

## Funding

This research is financially supported by the Ratchadapiseksompotch Fund, Graduate Affairs, Faculty of Medicine, Chulalongkorn University, Grant number GA68/039.

## Declaration of competing interests

The authors declare that they have no known competing financial interests or personal relationships that could have appeared to influence the work reported in this paper.

## Data Availability

The data supporting the conclusions of this article are included within the article and its supplementary files. The newly generated sequences were deposited in the GenBank database under the accession numbers PX169707-PX169727 (bats) and PV738151-PV738170 (*Eimeria* spp.).

## Appendix A Supplementary data

Supplementary Table S1 Information on 130 SSU rRNA sequences of *Eimeria* spp., from bats (128) and rodents (2), from Thailand and other geographical regions used for the haplotype network analysis. Supplementary Table S1

Supplementary Table S2 Information on 92 *Eimeria* spp. sequences and one *Toxoplasma gondii* sequence included in the phylogenetic analysis. Supplementary Table S2
