# Supplementary material for: New insights into molecular characterization and genetic diversity of Eimeria coccidian parasites in bats from diverse geographical regions of Thailand using nanopore-based DNA metabarcoding
Source: Curr Res Parasitol Vector Borne Dis. 2025 Oct 13;8:100327. doi: 10.1016/j.crpvbd.2025.100327 (PMC12557494; doi:10.1016/j.crpvbd.2025.100327)
Supplement: Supplementary Table S1 — Information on 130 SSU rRNA sequences of Eimeria spp., from bats (128) and rodents (2), from Thailand and other geographical regions used for the haplotype network analysis. [file mmc1.docx]

**Table S1**: Information on 130 SSU rRNA sequences of *Eimeria* spp., from bats (128) and rodents (2), from Thailand and other geographic regions used for the haplotype network analysis.

| **Accession number** | **Haplotype/Isolate/Species** | **Location** | **No. of sequences** |
| --- | --- | --- | --- |
| PV738151 | H01 | Chiang Rai, Thailand | 6 |
|  |  | Chonburi, Thailand | 4 |
|  |  | Chumphon, Thailand | 1 |
|  |  | Ratchaburi, Thailand | 1 |
|  |  | Uthai Thani, Thailand | 1 |
| PV738152 | H02 | Uthai Thani, Thailand | 1 |
| PV738153 | H03 | Bangkok, Thailand | 1 |
| PV738154 | H04 | Chonburi, Thailand | 11 |
|  |  | Chiang Rai, Thailand | 5 |
|  |  | Chumphon, Thailand | 3 |
|  |  | Bangkok, Thailand | 2 |
|  |  | Uthai Thani, Thailand | 2 |
| PV738155 | H05 | Chumphon, Thailand | 2 |
| PV738156 | H06 | Chonburi, Thailand | 1 |
| PV738157 | H07 | Bangkok, Thailand | 1 |
|  |  | Chiang Rai, Thailand | 1 |
| PV738158 | H08 | Chonburi, Thailand | 1 |
| PV738159 | H09 | Chiang Rai, Thailand | 6 |
|  |  | Chumphon, Thailand | 2 |
|  |  | Chonburi, Thailand | 1 |
|  |  | Uthai Thani, Thailand | 1 |
| PV738160 | H10 | Uthai Thani, Thailand | 1 |
| PV738161 | H11 | Uthai Thani, Thailand | 1 |
| PV738162 | H12 | Chumphon, Thailand | 1 |
| PV738163 | H13 | Chonburi, Thailand | 7 |
| PV738164 | H14 | Chiang Rai, Thailand | 1 |
| PV738165 | H15 | Ratchaburi, Thailand | 1 |
|  |  | Uthai Thani, Thailand | 2 |
| PV738166 | H16 | Uthai Thani, Thailand | 1 |
| PV738167 | H17 | Chiang Rai, Thailand | 1 |
| PV738168 | H18 | Chonburi, Thailand | 3 |
|  |  | Ratchaburi, Thailand | 4 |
| PV738169 | H19 | Chonburi, Thailand | 1 |
| PV738120 | H20 | Chiang Rai, Thailand | 1 |
| KC333453 | *Eimeria hessei* | France | 11 |
| AF307876 | *Eimeria antrozoi* | USA | 1 |
| AF324213 | *Eimeria catronensis* | USA | 1 |
| MH751961 | *Eimeria ferrisi* (rodent *Eimeria*) | Germany | 1 |
| KU192975 | *Eimeria jerfinica* (rodent *Eimeria*) | Czech Republic | 1 |
| MK284237 | *Eimeria macyi* | USA | 1 |
| AF324215 | *Eimeria pilarensis* | USA | 1 |
| AF307877 | *Eimeria rioarribaensis* | USA | 1 |
| LC371915 | *Eimeria* sp. Bat2 | Japan | 1 |
| LC089986 | *Eimeria* sp. Bat10 | Philippines | 1 |
| LC089983 | *Eimeria* sp. Bat31 | Philippines | 1 |
| MT813028 | *Eimeria* *jerfinica* M6 | Spain | 11 |
| MW182400 | *Eimeria* *rioarribaensis* M29 | Spain | 2 |
| MW182405 | *Eimeria* *jerfinica* M38 | Spain | 2 |
| MW182406 | *Eimeria* *jerfinica* M39 | Spain | 2 |
| OL588525 | *Eimeria* *jerfinica* M41 | Spain | 2 |
| MT598820 | *Eimeria* *rioarribaensis* M4 | Spain | 1 |
| MW182393 | *Eimeria* *rioarribaensis* M19 | Spain | 1 |
| MW182395 | *Eimeria* *jerfinica* M22 | Spain | 1 |
| MW182396 | *Eimeria* *jerfinica* M24 | Spain | 1 |
| MW182397 | *Eimeria* *jerfinica* M25 | Spain | 1 |
| MW182398 | *Eimeria* *jerfinica* M27 | Spain | 1 |
| MW182399 | *Eimeria* *jerfinica* M28 | Spain | 1 |
| MW182401 | *Eimeria* *jerfinica* M30 | Spain | 1 |
| MW182402 | *Eimeria* *jerfinica* M31 | Spain | 1 |
| MW182403 | *Eimeria* *jerfinica* M32 | Spain | 1 |
| MT338554 | *Eimeria* sp. Saudi Arabia | Saudi Arabia | 1 |
