# Supplementary material for: New insights into molecular characterization and genetic diversity of Eimeria coccidian parasites in bats from diverse geographical regions of Thailand using nanopore-based DNA metabarcoding
Source: Curr Res Parasitol Vector Borne Dis. 2025 Oct 13;8:100327. doi: 10.1016/j.crpvbd.2025.100327 (PMC12557494; doi:10.1016/j.crpvbd.2025.100327)
Supplement: Supplementary Table S2 — Information on 92 Eimeria spp. sequences and one Toxoplasma gondii sequence included in the phylogenetic analysis. [file mmc2.docx]

**Table S2**: The information on 92 *Eimeria* spp. sequences and one *Toxoplasma gondii* sequence included in the phylogenetic analysis.

| **Accession number** | **Species** | **Host** | **Country** | **References** |
| --- | --- | --- | --- | --- |
| U67115 | *Eimeria acervulina* | Chicken  (*Gallus gallus*) | USA | Barta et al., 1997 |
| AF291427 | *Eimeria alabamensis* | Not identified  (Previously reported in cattle (*Bos taurus*)) | Sweden | Svensson et al., 1994; Ellis et al., 2000 |
| AF307880 | *Eimeria albigulae* | White-throated woodrat  (*Neotoma albigula*) | USA | Zhao et al., 2001 |
| JQ993659 | *Eimeria alorani* | Striped field mouse  (*Apodemus agrarius*) | Slovakia | Kvičerová and Hypša, 2013 |
| AF307876 | *Eimeria antrozoi* | Pallid bat  (*Antrozous pallidus*) | USA | Duszynski et al., 1999; Zhao et al., 2001 |
| KU192945 | *Eimeria apionodes* | Yellow-necked mouse  (*Apodemus flavicollis*) | Italy | Mácová et al., 2018 |
| AF307878 | *Eimeria arizonensis* | Deer mouse  (*Peromyscus maniculatus*) | USA | Zhao et al., 2001 |
| AB769596 | *Eimeria bukidnonensis* | Cattle  (*Bos taurus*) | Japan | Kokuzawa et al., 2013 |
| JQ993666 | *Eimeria burdai* | Silvery mole-rat  (*Heliophobius argenteocinereus*) | Czech Republic | Kvičerová and Hypša, 2013 |
| JQ993646 | *Eimeria cahirinensis* | Eastern spiny mouse  (*Acomys dimidiatus*) | Israel | Kvičerová and Hypša, 2013 |
| JQ993648 | *Eimeria callospermophili* | European ground squirrel  (*Spermophilus citellus*) | Czech Republic | Kvičerová and Hypša, 2013 |
| AF324213 | *Eimeria catronensis* | Not identified  (Previously reported in little brown bat  (*Myotis lucifugus*)) | USA | Unpublished |
| JQ993649 | *Eimeria caviae* | Guinea pig  (*Cavia porcellus*) | Czech Republic | Kvičerová and Hypša, 2013 |
| AF339489 | *Eimeria chaetodipi* | Hispid pocket mouse  (*Chaetodipus hispidus*) | USA | Zhao and Duszynski, 2001 |
| JQ993650 | *Eimeria chinchillae* | Long-tailed chinchilla  (*Chinchilla lanigera*) | Czech Republic | Kvičerová and Hypša, 2013 |
| AF324214 | *Eimeria chobotari* | Merriam’s kangaroo rat  (*Dipodomys merriami*) | USA | Zhao and Duszynski, 2001 |
| HQ173828 | *Eimeria coecicola* | European rabbit  (*Oryctolagus cuniculus*) | Czech Republic | Unpublished |
| AF339490 | *Eimeria dipodomysis* | Phillips’s kangaroo rat  (*Dipodomys phillipsii*) | USA | Zhao and Duszynski, 2001 |
| AF080614 | *Eimeria falciformis* | Not identified  (Previously reported in house mouse (*Mus musculus*)) | Canada | Barta et al., 2001 |
| MH751961 | *Eimeria ferrisi* | House mouse  (*Mus musculus*) | Germany | Jarquín-Díaz et al., 2019 |
| KC333453 | *Eimeria hessei* | Lesser horseshoe bat  (*Rhinolophus hipposideros*) | France | Afonso et al., 2014 |
| KU192975 | *Eimeria jerfinica* | Wood mouse  (*Apodemus sylvaticus*) | Czech Republic | Mácová et al., 2018 |
| KU192917 | *Eimeria kaunensis* | Yellow-necked mouse  (*Apodemus flavicollis*) | Czech Republic | Mácová et al., 2018 |
| AB849922 | *Eimeria krijgsmanni* | House mouse  (*Mus musculus*) | Japan | Takeo et al., 2014 |
| ON476843 | *Eimeria lancasterensis* | Eastern gray squirrel  (*Sciurus carolinensis*) | USA | McAllister et al., 2024 |
| AF311640 | *Eimeria langebarteli* | Western harvest mouse  (*Reithrodontomys megalotis*) | USA | Zhao and Duszynski, 2001 |
| AF339491 | *Eimeria leucopi* | White-footed mouse  (*Peromyscus leucopus*) | USA | Zhao and Duszynski, 2001 |
| MK284237 | *Eimeria macyi* | Eastern red bat  (*Lasiurus borealis*) | USA | Miles et al., 2019 |
| JF304148 | *Eimeria myoxi* | Garden dormouse  (*Eliomys quernicus*) | Czech Republic | Kvičerová et al., 2011 |
| JQ993665 | *Eimeria nafuko* | Silvery mole-rat  (*Heliophobius argenteocinereus*) | Czech Republic | Kvičerová and Hypša, 2013 |
| U40263 | *Eimeria nieschulzi* | Not identified  (Previously reported in brown rat (*Rattus norvegicus*)) | USA | Relman et al., 1996 |
| ON476844 | *Eimeria ontarioensis* | Eastern gray squirrel  (*Sciurus carolinensis*) | USA | McAllister et al., 2024 |
| AF307879 | *Eimeria onychomysis* | Northern grasshopper mouse  (*Onychomys leucogaster*) | USA | Hnida and Duszynski, 1999; Zhao et al., 2001 |
| KT184350 | *Eimeria papillata* | House mouse  (*Mus musculus*) | USA | Ogedengbe et al., 2016 |
| AF324215 | *Eimeria pilarensis* | Not identified  (Previously reported in western small-footed bat  (*Myotis ciliolabrum*)) | USA | Unpublished |
| AF339492 | *Eimeria peromysci* | Pinyon mouse  (*Peromyscus truei*) | USA | Zhao and Duszynski, 2001 |
| AF311642 | *Eimeria reedi* | Silky pocket mouse  (*Perognathus flavus*) | USA | Zhao and Duszynski, 2001 |
| AF307877 | *Eimeria rioarribaensis* | Western small-footed bat  (*Myotis ciliolabrum*) | USA | Duszynski et al., 1999; Zhao et al., 2001 |
| MN650661 | *Eimeria sciurorum* | Calabrian black squirrel  (*Sciurus meridionalis*) | Italy | Kvicerova et al., 2020 |
| AF324216 | *Eimeria scholtysecki* | Agile kangaroo rat  (*Dipodomys agilis*) | USA | Zhao and Duszynski, 2001 |
| AF311643 | *Eimeria separata* | Brown rat  (*Rattus norvegicus*) | USA | Zhao and Duszynski, 2001 |
| AF311644 | *Eimeria sevilletensis* | Mearns’s grasshopper mouse  (*Onychomys arenicola*) | USA | Zhao and Duszynski, 2001 |
| HQ173837 | *Eimeria stiedai* | European rabbit  (*Oryctolagus cuniculus*) | Czech Republic | Unpublished |
| KT184353 | *Eimeria tamiasciuri* | American red squirrel  (*Tamiasciurus hudsonicus*) | Canada | Ogedengbe et al., 2016 |
| AF246717 | *Eimeria telekii* | Typical striped grass mouse  (*Lemniscomys striatus*) | Kenya | Šlapeta et al., 2001 |
| KT184354 | *Eimeria tenella* | Chicken  (*Gallus gallus*) | USA | Ogedengbe et al., 2016 |
| KU192941 | *Eimeria uptoni* | Yellow-necked mouse  (*Apodemus flavicollis*) | Poland | Mácová et al., 2018 |
| MH751957 | *Eimeria vermiformis* | House mouse  (*Mus musculus*) | Germany | Jarquín-Díaz et al., 2019 |
| JQ993653 | *Eimeria vilasi* | Wyoming ground squirrel  (*Spermophilus elegans*) | USA | Kvičerová and Hypša, 2013 |
| MG770470 | *Eimeria* sp. 34RUM | White-toothed shrew  (*Crocidura* sp.) | Romania | Unpublished |
| MG770465 | *Eimeria* sp. 54STR | Lesser white-toothed shrew  (*Crocidura suaveolens*) | Czech Republic | Unpublished |
| MG770469 | *Eimeria* sp. B4B2 | White-toothed shrew  (*Crocidura* sp.) | Bulgaria | Unpublished |
| LC371915 | *Eimeria* sp. Bat2 | Northern bat  (*Eptesicus nilssonii*) | Japan | Murakoshi et al., 2018 |
| LC089986 | *Eimeria* sp. Bat10 | Philippine forest horseshoe bat  (*Rhinolophus inops*) | Philippines | Murakoshi et al., 2016 |
| LC089983 | *Eimeria* sp. Bat31 | Cave nectar bat  (*Eonycteris spelaea*) | Philippines | Murakoshi et al., 2016 |
| PV738151 | *Eimeria* sp. H01 | Cave nectar bat (*E. spelaea*), great roundleaf bat (*Hipposideros armiger*), *H. larvatus* species complex, Lyle’s flying fox (*Pteropus lylei*), black-bearded tomb bat (*Taphozous melanopogon*) | Thailand | In this study |
| PV738152 | *Eimeria* sp. H02 | Black-bearded tomb bat  (*T. melanopogon*) | Thailand | In this study |
| PV738153 | *Eimeria* sp. H03 | Lesser short-nosed fruit bat  (*Cynopterus brachyotis*) | Thailand | In this study |
| PV738154 | *Eimeria* sp. H04 | Lesser short-nosed fruit bat (*C. brachyotis*), cave nectar bat (*E. spelaea*), *H. larvatus* species complex, Lyle’s flying fox (*P. lylei*), large flying fox (*P. vampyrus*), black-bearded tomb bat (*T. melanopogon*) | Thailand | In this study |
| PV738155 | *Eimeria* sp. H05 | Black-bearded tomb bat  (*T. melanopogon*) | Thailand | In this study |
| PV738156 | *Eimeria* sp. H06 | Unknown host | Thailand | In this study |
| PV738157 | *Eimeria* sp. H07 | Lesser short-nosed fruit bat  (*C. brachyotis*) | Thailand | In this study |
| PV738158 | *Eimeria* sp. H08 | Large flying fox  (*P. vampyrus*) | Thailand | In this study |
| PV738159 | *Eimeria* sp. H09 | Cave nectar bat (*E. spelaea*), Lyle’s flying fox (*P. lylei*), black-bearded tomb bat (*T. melanopogon*) | Thailand | In this study |
| PV738160 | *Eimeria* sp. H10 | Unknown host | Thailand | In this study |
| PV738161 | *Eimeria* sp. H11 | Black-bearded tomb bat  (*T. melanopogon*) | Thailand | In this study |
| PV738162 | *Eimeria* sp. H12 | Black-bearded tomb bat  (*T. melanopogon*) | Thailand | In this study |
| PV738163 | *Eimeria* sp. H13 | Lyle’s flying fox  (*P. lylei*) | Thailand | In this study |
| PV738164 | *Eimeria* sp. H14 | Unknown host | Thailand | In this study |
| PV738165 | *Eimeria* sp. H15 | Great roundleaf bat (*H. armiger*), *H. larvatus* species complex | Thailand | In this study |
| PV738166 | *Eimeria* sp. H16 | Great roundleaf bat (*H. armiger*), *H. larvatus* species complex | Thailand | In this study |
| PV738167 | *Eimeria* sp. H17 | Unknown host | Thailand | In this study |
| PV738168 | *Eimeria* sp. H18 | Wrinkle-lipped free-tailed bat (*Mops plicatus*), Lyle’s flying fox (*P. lylei*) | Thailand | In this study |
| PV738169 | *Eimeria* sp. H19 | Large flying fox  (*P. vampyrus*) | Thailand | In this study |
| PV738170 | *Eimeria* sp. H20 | Cave nectar bat (*E. spelaea*), black-bearded tomb bat (*T. melanopogon*) | Thailand | In this study |
| MT598820 | *Eimeria rioarribaensis* M4 | Mediterranean horseshoe bat  (*Rhinolophus euryale*) | Spain | Couso-Pérez et al., 2022 |
| MT813028 | *Eimeria jerfinica* M6 | Greater noctule bat  (*Nyctalus lasiopterus*) | Spain | Couso-Pérez et al., 2022 |
| MW182393 | *Eimeria rioarribaensis* M19 | Greater noctule bat  (*Nyctalus lasiopterus*) | Spain | Couso-Pérez et al., 2022 |
| MW182395 | *Eimeria jerfinica* M22 | Leisler's bat  (*Nyctalus leisleri*) | Spain | Couso-Pérez et al., 2022 |
| MW182396 | *Eimeria jerfinica* M24 | Daubenton’s bat  (*Myotis daubentonii*) | Spain | Couso-Pérez et al., 2022 |
| MW182397 | *Eimeria jerfinica* M25 | Common pipistrelle bat  (*Pipistrellus pipistrellus*) | Spain | Couso-Pérez et al., 2022 |
| MW182398 | *Eimeria jerfinica* M27 | Serotine bat  (*Eptesicus serotinus*) | Spain | Couso-Pérez et al., 2022 |
| MW182399 | *Eimeria jerfinica* M28 | Long-fingered bat  (*Myotis capaccinii*) | Spain | Couso-Pérez et al., 2022 |
| MW182400 | *Eimeria rioarribaensis* M29 | Greater mouse-eared bat  (*Myotis myotis*) | Spain | Couso-Pérez et al., 2022 |
| MW182401 | *Eimeria jerfinica* M30 | Common pipistrelle bat  (*Pipistrellus pipistrellus*) | Spain | Couso-Pérez et al., 2022 |
| MW182402 | *Eimeria jerfinica* M31 | Savi’s pipistrelle bat  (*Hypsugo savii*) | Spain | Couso-Pérez et al., 2022 |
| MW182403 | *Eimeria jerfinica* M32 | Isabelline serotine bat  (*Eptesicus isabellinus*) | Spain | Couso-Pérez et al., 2022 |
| MW182405 | *Eimeria jerfinica* M38 | Schreiber's bat  (*Miniopterus schreibersii*) | Spain | Couso-Pérez et al., 2022 |
| MW182406 | *Eimeria jerfinica* M39 | Lesser mouth-eared bat  (*Myotis blythii*) | Spain | Couso-Pérez et al., 2022 |
| OL588525 | *Eimeria jerfinica* M41 | Greater mouse-eared bat  (*Myotis myotis*) | Spain | Couso-Pérez et al., 2022 |
| MT338554 | *Eimeria* sp. Saudi Arabia | White-bellied yellow bat  (*Scotophilus leucogaster*) | Saudi Arabia | Mohammed et al., 2020 |
| MH349726 | *Eimeria* sp. ex *Tarsius syrichta* | Philippine tarsier  (*Carlito syrichta*) | Philippines | Hofmannová et al., 2018 |
| M97703 | *Toxoplasma gondii* | Not identified | Not identified | Johnson et al., 1987; Guay et al., 1992; Gagnon et al., 1993 |

**References**

Afonso, E., Baurand, P.E., Tournant, P., Capelli, N., 2014. First amplification of *Eimeria hessei* DNA from the lesser horseshoe bat (*Rhinolophus hipposideros*) and its phylogenetic relationships with *Eimeria* species from other bats and rodents. Exp. Parasitol. 139, 58-62.

Barta, J.R., Martin, D.S., Carreno, R.A., Siddall, M.E., Profous-Juchelkat, H., Hozza, M., et al., 2001. Molecular phylogeny of the other tissue coccidia: Lankesterella and Caryospora. J. Parasitol. 87, 121-127.

Barta, J.R., Martin, D.S., Liberator, P.A., Dashkevicz, M., Anderson, J.W., Feighner, S.D., et al., 1997. Phylogenetic relationships among eight *Eimeria* species infecting domestic fowl inferred using complete small subunit ribosomal DNA sequences. J. Parasitol. 83, 262-271.

Couso-Pérez, S., Pardavila, X., Ares-Mazás, E., Gómez-Couso, H., 2022. Molecular identification of *Eimeria* species in Spanish bats. Parasitol. Int. 91, 102621.

Duszynski, D.W., Scott, D.T., Aragon, J., Leach, A., Perry, T., 1999. Six new *Eimeria* species from vespertilionid bats of North America. J. Parasitol. 85, 496-503.

Ellis, J.T., Holmdahl, O.J.M., Ryce, C., Njenga, J.M., Harper, P.A.W., Morrison, D.A., 2000. Molecular Phylogeny of *Besnoitia* and the Genetic Relationships Among *Besnoitia* of Cattle, Wildebeest and Goats. Protist 151, 329-336.

Gagnon, S., Levesque, R.C., Sogin, M.L., Gajadhar, A.A., 1993. Molecular cloning, complete sequence of the small subunit ribosomal RNA coding region and phylogeny of *Toxoplasma gondii*. Mol. Biochem. Parasitol. 60, 145-148.

Guay, J.M., Huot, A., Gagnon, S., Tremblay, A., Levesque, R.C., 1992. Physical and genetic mapping of cloned ribosomal DNA from *Toxoplasma gondii*: primary and secondary structure of the 5S gene. Gene 114, 165-171.

Hnida, J.A., Duszynski, D.W., 1999. Cross-Transmission Studies with *Eimeria arizonensis*, *E. arizonensis*-like Oocysts and *Eimeria langebarteli*: Host Specificity at the Genus and Species Level within the Muridae. J. Parasitol. 85, 873-877.

Hofmannová, L., Jirků, M., Řeháková, M., Kvičerová, J., 2018. Two new species of *Eimeria* (Apicomplexa: Eimeriidae) in Philippine tarsier (*Tarsius syrichta*). Eur. J. Protistol. 66, 77-85.

Jarquín-Díaz, V.H., Balard, A., Jost, J., Kraft, J., Dikmen, M. N., Kvičerová, J., et al., 2019. Detection and quantification of house mouse *Eimeria* at the species level – Challenges and solutions for the assessment of coccidia in wildlife. Int. J. Parasitol. Parasites Wildl. 10, 29-40.

Johnson, A.M., Murray, P.J., Illana, S., Baverstock, P.J., 1987. Rapid nucleotide sequence analysis of the small subunit ribosomal RNA of *Toxoplasma gondii*: evolutionary implications for the Apicomplexa. Mol. Biochem. Parasitol. 25, 239-246.

Kokuzawa, T., Ichikawa-Seki, M., Itagaki, T., 2013. Determination of phylogenetic relationships among *Eimeria* species, which parasitize cattle, on the basis of nuclear 18S rDNA sequence. J. Vet. Med. Sci. 75, 1427-1431.

Kvičerová, J., Hofmannová, L., Scognamiglio, F., Santoro, M., 2020. *Eimeria sciurorum* (Apicomplexa, Coccidia) From the Calabrian Black Squirrel (*Sciurus meridionalis*): An Example of Lower Host Specificity of Eimerians. Front. Vet. Sci. 7, 369.

Kvičerová, J., Hypša, V., 2013. Host-Parasite Incongruences in Rodent *Eimeria* Suggest Significant Role of Adaptation Rather than Cophylogeny in Maintenance of Host Specificity. PLoS One 8, e63601.

Kvičerová, J., Mikeš, V., Hypša, V., 2011. Third lineage of rodent eimerians: morphology, phylogeny and re-description of *Eimeria myoxi* (Apicomplexa: Eimeriidae) from *Eliomys quercinus* (Rodentia: Gliridae). Parasitology 138, 1217-1223.

Mácová, A., Hoblíková, A., Hypša, V., Stanko, M., Martinů, J., Kvičerová, J., 2018. Mysteries of host switching: Diversification and host specificity in rodent-coccidia associations. Mol. Phylogenet. Evol. 127, 179-189.

McAllister, C.T., Motriuk-Smith, D., McCurdy, H., Seville, R., 2024. Characterization of *Eimeria lancasterensis* and *Eimeria ontarioensis* (Apicomplexa: Eimeriidae) Found in Eastern Gray Squirrels, *Sciurus carolinensis* (Rodentia: Sciuridae), from Arkansas and Oklahoma. J. Parasitol. 110, 90-95.

Miles, T.P., Rush, S.A., Rosser, T.G., 2019. Morphological, molecular and phylogenetic characterisation of *Eimeria macyi* Wheat, 1975 (Apicomplexa: Eimeriidae) in the eastern red bat *Lasiurus borealis* (Müller) from Mississippi, USA. Syst. Parasitol. 96, 245-255.

Mohammed, O.B., Duszynski, D.W., Amor, N., Alagaili, A.N., 2020. A novel coccidian (Apicomplexa: Eimeriidae) from *Scotophilus leucogaster* (Chiroptera: Vespertilionidae) in southern Saudi Arabia. Parasitol. Res. 119, 3845-3852.

Murakoshi, F., Koyama, K., Akasaka, T., Horiuchi, N., Kato, K., 2018. Molecular and histopathological characterization of *Cryptosporidium* and *Eimeria* species in bats in Japan. J. Vet. Med. Sci. 80, 1395-1399.

Murakoshi, F., Recuenco, F.C., Omatsu, T., Sano, K., Taniguchi, S., Masangkay, J.S., et al., 2016. Detection and molecular characterization of *Cryptosporidium* and *Eimeria* species in Philippine bats. Parasitol. Res. 115, 1863-1869.

Ogedengbe, M.E., Ogedengbe, J.D., Whale, J.C., Elliot, K., Juárez-Estrada, M. A., Barta, J.R., 2016. Molecular phylogenetic analyses of tissue coccidia (sarcocystidae; apicomplexa) based on nuclear 18s rDNA and mitochondrial COI sequences confirms the paraphyly of the genus *Hammondia*. Parasitol. Open 2, e2.

Relman, D.A., Schmidt, T.M., Gajadhar, A., Sogin, M., Cross, J., Yoder, K., et al., 1996. Molecular phylogenetic analysis of *Cyclospora*, the human intestinal pathogen, suggests that it is closely related to *Eimeria* species. J. Infect. Dis., 173, 440-445.

Šlapeta, J.R., Modry, D., Votýpka, J., Jirků, M., Oborník, M., Lukeš Jr, J., et al., 2001. *Eimeria telekii* n.sp. (Apicomplexa: Coccidia) from *Lemniscomys striatus* (Rodentia: Muridae): Morphology, pathology and phylogeny. Parasitology 122, 133-143.

Svensson, C., Uggla, A., Pehrson, B., 1994. *Eimeria alabamensis* infection as a cause of diarrhoea in calves at pasture. Vet. Parasitol. 53, 33-43.

Takeo, T., Tanaka, T., Matsubayashi, M., Maeda, H., Kusakisako, K., Matsui, T., et al., 2014. Molecular and phylogenetic characterizations of an *Eimeria krijgsmanni* Yakimoff & Gouseff, 1938 (Apicomplexa: Eimeriidae) mouse intestinal protozoan parasite by partial 18S ribosomal RNA gene sequence analysis. Parasitol. Int. 63, 627-630.

Zhao, X., Duszynski, D.W., 2001. Molecular phylogenies suggest the oocyst residuum can be used to distinguish two independent lineages of *Eimeria* spp in rodents. Parasitol. Res. 87, 638-643.

Zhao, X., Duszynski, D.W., Loker, E.S., 2001. Phylogenetic position of *Eimeria antrozoi*, a bat coccidium (Apicomplexa: Eimeriidae) and its relationship to morphologically similar Eimeria spp. from bats and rodents based on nuclear 18S and plastid 23S rDNA sequences. J. Parasitol., 87(5), 1120-1123.
